# Aberrant Splicing in *GJB1* and the Relevance of 5′ UTR in CMTX1 Pathogenesis

**DOI:** 10.3390/brainsci11010024

**Published:** 2020-12-27

**Authors:** Federica Boso, Federica Taioli, Ilaria Cabrini, Tiziana Cavallaro, Gian Maria Fabrizi

**Affiliations:** 1 Department of Neurological Sciences, Biomedicine and Movement Sciences, University of Verona, Piazzale L.A. Scuro 10, 37134 Verona, Italy; federica.boso-1@unitn.it (F.B.); federica.taioli@univr.it (F.T.); ilaria.cabrini@univr.it (I.C.); 2 Department of Cellular, Computational and Integrative Biology, University of Trento, Via Sommarive 9, 38123 Povo (Trento), Italy; 3 Azienda Ospedaliera Universitaria Integrata Verona—Borgo Roma, Piazzale L.A. Scuro 10, 37134 Verona, Italy; tiziana.cavallaro@aovr.veneto.it

**Keywords:** Charcot-Marie-Tooth, CMT, X-linked Charcot-Marie-Tooth (CMTX1), Connexin 32, *GJB1*, 5′ UTR, noncoding, splicing

## Abstract

The second most common form of Charcot-Marie-Tooth disease (CMT) follows an X-linked dominant inheritance pattern (CMTX1), referring to mutations in the gap junction protein beta 1 gene (*GJB1*) that affect connexin 32 protein (Cx32) and its ability to form gap junctions in the myelin sheath of peripheral nerves. Despite the advances of next-generation sequencing (NGS), attention has only recently also focused on noncoding regions. We describe two unrelated families with a c.-17+1G>T transversion in the 5′ untranslated region (UTR) of *GJB1* that cosegregates with typical features of CMTX1. As suggested by in silico analysis, the mutation affects the regulatory sequence that controls the proper splicing of the intron in the corresponding mRNA. The retention of the intron is also associated with reduced levels of the transcript and the loss of immunofluorescent staining for Cx32 in the nerve biopsy, thus supporting the hypothesis of mRNA instability as a pathogenic mechanism in these families. Therefore, our report corroborates the role of 5′ UTR of *GJB1* in the pathogenesis of CMTX1 and emphasizes the need to include this region in routine *GJB1* screening, as well as in NGS panels.

## 1. Introduction

Charcot-Marie-Tooth disease (CMT) refers to the most frequent group of hereditary neuropathies, encompassing a wide range of genetic, clinical, neurophysiological and pathological features. Despite significant genetic heterogeneity, most known mutations involve four genes (*PMP22*, *GJB1*, *MFN2* and *MPZ*) [1,2], but, until recently, molecular diagnosis was hampered by a costly and gruelling search for those main causative genes by multiplex ligation-dependent probe amplification (MLPA) and conventional Sanger sequencing using a candidate-gene approach. Indeed, the advent of next-generation sequencing (NGS) techniques paved the way for a broader screening of the patients and for the discovery of rarer variants, thus providing a higher likelihood of identification. Notwithstanding this considerable progress, assuming a Mendelian inheritance, a genetic diagnosis remains elusive in 30–70% of CMT patients [1,2,3,4], depending on customized gene panels that generally cover only known disease-related coding sequences in order to provide a faster and affordable analysis with higher coverage, as well as fewer incidental findings. As an example, the most frequent culprit for CMT after peripheral myelin protein 22 (*PMP22)* is the gap junction beta 1 protein gene (*GJB1*), affecting about 6.5–17% of the patients with a presumptive inherited neuropathy [2,3]. Human *GJB1* consists of two exons, separated by an intron of variable size: exon 1 encodes most of the 5′ untranslated region (UTR), whereas exon 2 encompasses the entire amino acid coding region and the 3′ UTR. Two different tissue-specific promoters have been acknowledged [5]: a basal promoter P1 is located more than 8 kb upstream from the coding sequence and regulates the transcript NM_001097642 in liver, pancreas, oocytes and embryonic stem cells, while in the peripheral nervous system, the alternative promoter P2 (the 130-bp exon 1B, which is separated from exon 2 by the 356-bp intron 1B) is responsible for the production of the transcript NM_000166. By alternative promoter usage, *GJB1* thus provides mRNAs with identical coding regions but different 5′ UTR with specific cis-regulatory elements (Supplementary Figure S1). Thanks to the broader availability of genetic screening, today there are over 450 known variants in the coding sequence, with a majority of missense mutations and rarer cases with frameshift and premature stop codon mutations or ample deletions. However, noncoding regions are increasingly recognized not only as key regulators of protein expressions, but also as potential hidden causes of diseases, accounting for so much as 10% of patients with *GJB1* mutations [3]. Indeed, variations of the UTR may become pathogenic by disrupting the sequences that regulate transcription (such as binding sites for transcription factors like Early Growth Response-2, EGR2 and SRY-Box 10, SOX10) or by impairing mRNA translation and stability, thus influencing protein expression [6,7].

We propose the candidate variant c.-17+1G>T as an example of a 5′ UTR mutation that may account for mRNA instability by aberrant splicing of intron 1B, ultimately causing CMTX1.

## 2. Patients, Materials and Methods

**Figure 1:**
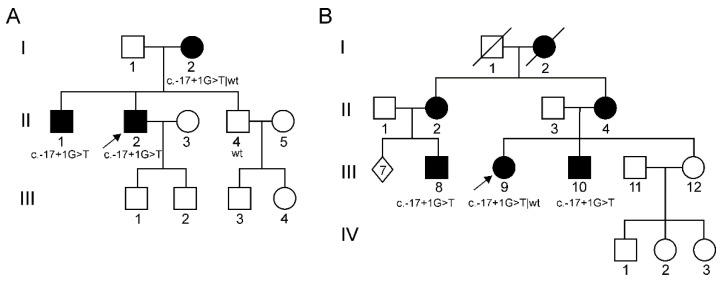
Pedigree tree of both families. (**A**) First family; (**B**) Second family. Arrows indicate the probands. Black circle/square: female-/male-affected individuals; open circle/square: female/male healthy individuals; wt = wild-type. LOD (logarithm of the odds) score was 2.107, assuming an X-linked dominant model of inheritance.

Two young probands who were evaluated for a length-dependent, sensory-motor neuropathy led to the study of two unrelated families on the assumption of a genetic pathogenesis (Figure 1A, II-2; Figure 1B, III-9). Both cases presented with typical features of CMT, reporting walking difficulties, sensory abnormalities and slowly progressive distal muscle weakness with peroneal atrophy and foot deformities. The male proband also had further clues such as early onset (in the second decade), split hand syndrome, postural tremor and bilateral hypoacusia. Likewise, pedigree analysis revealed similarly affected relatives (Table 1), with a tendency for males to be more severely and prematurely affected and with no cases of male-to-male transmission, thus suggesting an X-linked dominant inheritance pattern.

### 2.1. Mutational Analysis

Genomic DNA was extracted from blood samples of individuals of both families after receiving written informed consent for genetic testing, according to the local Ethical Committed procedures. MLPA ruled out copy number variations of *PMP22*, *GJB1* and *MPZ*. Mutational analysis, done before the advent of NGS era, was performed by denaturing high-performance liquid chromatography (DHPLC) (Wave^®^ System 3500 HT Transgenomic, Transgenomic Inc., Omaha, NE, USA) and automated nucleotide sequencing (CEQ 8800 Beckman automated sequencer, Beckman Coulter Inc., Brea, CA, USA), as previously described [8]. The screening targeted the following CMT-associated genes: *GJB1*, *MPZ*, *PMP22*, *NEFL*, *MFN2*, *EGR2*, *GDAP1*, *HSPB1*, *HSPB8*, *GARS*, *YARS*, *BSCL2*, *DNM2* and *TRPV4.* Analysis of *GJB1* (NM_000166; NG_008357.1) included the coding region (exon 2), as well as 5′ UTR (from c.-146-324 to c.1) and 108 nt of the 3′ UTR sequence.

Our DNA biobank was approved by the institutional ethic committee (Comitato etico per la Sperimentazione Clinica, CESC) on 11/02/2015 (project identification code BIOB-NEU-DNA-2014, protocol 13582, 20 March 2015). Mutations were reported according to the latest Human Genome Variation Society (HGVS) nomenclature. Variant interpretation was performed by Ensembl Variant Effect Predictor (threshold value = 0.6) [9], while NNSplice and NetGene2 algorithms were used to predict splicing sites in the DNA. 

### 2.2. Transcriptional Analysis

Total RNA was extracted (TRI Reagent kit^®^, Ambion, Austin, TX, USA) from archived frozen sural nerve biopsies of patient III-8 (second family), using data from a 54-year-old male patient affected by Chronic Inflammatory Demyelinating Polyradiculoneupathy (CIDP) as control. After treatment with DNase I (Sigma-Aldrich, St.Louis, MO, USA) to avoid genomic DNA contamination, RNA was retrotranscribed using random hexamer primers (ThermoScriptTM RT-PCR System, Invitrogen, Carlsbad, CA, USA). To analyse RNA splicing, cDNA was then amplified by real-time polymerase chain reaction (RT-PCR) using primers spanning exon 1 B and the first 23 nucleotides of exon 2. RT-PCR was also applied with primers spanning *GJB1* exon 2. The amplified products were sequenced as previously described, and differences in splice variants were visualized and approximately quantified using ImageQuant System 5.2 (GE Healthcare, Chicago, IL, USA) on agarose gel electrophoretic bands. As references, primers were also designed to amplify the housekeeping *GAPDH* and myelin related *PMP22*. Further methodological details are available as supplementary materials (Supplementary Table S1).

### 2.3. Immunofluorescence Study

Longitudinal cryostatic nerve sections were used for immunofluorescence staining. After incubation with mouse monoclonal antibody directed against amino acids 95–125 in the central cytoplasmic loop of rat Cx32 (dilution 1:200; Chemicon International Inc., Temecula, CA, USA), biotinylated anti-mouse IgG antibodies (Amersham Pharmacia Biotech, Piscataway, NJ, USA, 1:100) along with Texas red (Vector Laboratories, Burlingame, CA, USA, 1:100) were applied to detect Cx32. Serum from a patient with anti-myelin associated glycoprotein (MAG) neuropathy (anti-MAG autoantibodies titer = 258,000 Bühlmann Titer Units, according to ELISA quantitative determination, Bühlmann laboratories) was used as the primary antibody (1:200) to identify paranodes and Schmidt-Lanterman incisures by rabbit anti-human fluorescein-conjugated IgM labelling (DakoCytomation, Carpinteria, CA, USA, 1:200). Nerve sections were then analysed with confocal laser-scanning microscope (Zeiss LSM 510, argon 418 lambda and helio-neon 543 lambda laser, Oberkochen, Germany), simultaneously capturing fluorescent signals from both channels (Cx32 appearing in red and MAG in green) to obtain merged images and highlight colocalization. Negative controls used only secondary antibodies.

## 3. Results

### 3.1. Patients

**Figure 2:**
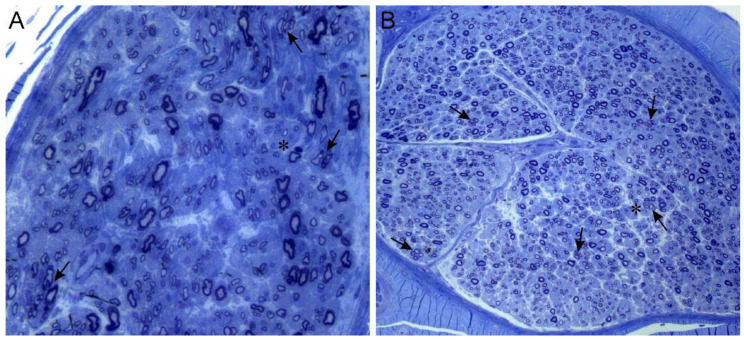
Sural nerve biopsies. Semithin sections stained with toluidin blue. (**A**) (20×): 17-year-old patient II-2, first family. (**B**) (10×): 18-year-old patient III-8, second family. Both biopsies show mild loss of large myelinated fibres, several clusters of regeneration (arrows) and few simple onion bulbs (asterisk).

Clinical features of the affected members of both families (Table 1) were consistent with CMTX1. Although no systematic investigations by magnetic resonance imaging (MRI) were performed, no significant clinical signs of central nervous system involvement were reported. On the other hand, several members of the families underwent further diagnostic procedures to investigate the symptoms concerning the peripheral nervous system. Conventional nerve conduction studies showed both axonal damage and demyelinating features in all cases. Three male patients (II-2 in Figure 1A; III-8 and III-10 in Figure 1B) also underwent a sural biopsy in their adolescence, disclosing a severe reduction of myelinated fibre density and sparse regeneration clusters (Figure 2). 

### 3.2. Mutational Analysis

Molecular genetic analysis excluded pathogenic mutations in a gene panel including the coding sequence of *GJB1* as well as other typical CMT genes. However, all affected members of both families had a c.-17+1G>T transversion in the 5′ UTR of *GJB1*, thus identifying hemizygous males and heterozygous females with the same mutation, which was absent in male healthy relatives (Supplementary Figure S2).

In silico analysis predicted its pathogenic relevance, as this variant involves a phylogenetically conserved nucleotide and is supposed to cancel a canonical donor splice site in intron 1B (ADA score and RF score were, respectively, 0.999 and 0.842). The variant was absent from gnomAD database and was predicted to be pathogenic according to ACGM criteria [10].

### 3.3. Transcriptional Analysis

**Figure 3:**
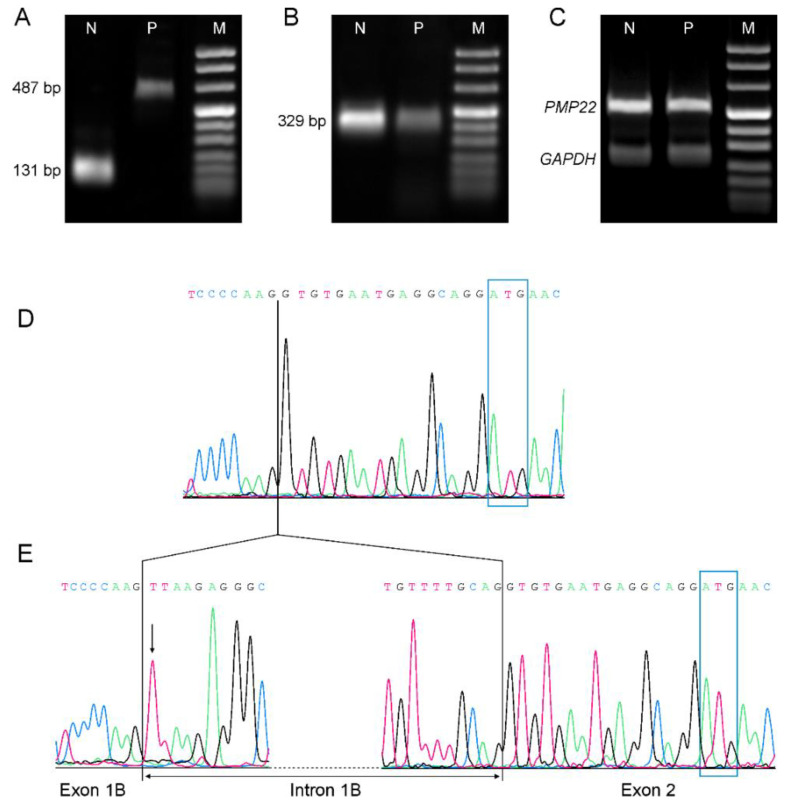
Comparative transcriptional and retro-transcriptional analysis. (**A**–**C**): Real-time polymerase chain reaction (RT-PCR) of mRNA extracted from archived frozen sural nerve biopsies of a normal control (N) and patient III-8 (P), separated by 2% agarose. (**D**,**E**): Nucleotide sequence of *GJB1* cDNA, as obtained from sural nerve biopsy of a normal control (**D**) and patient III-8 (**E**). ATG start codon is outlined by the blue rectangles. (**A**) Analysis of *GJB1* RNA splicing using primers spanning exon 1B and the first 23 nucleotides of exon 2 (from g.13017 to g.13503): N shows an amplicon of 131 bp, as expected after regular mRNA splicing. In P, the same amplicon has an estimated length of 487 pb, which is consistent with the inclusion of intron 1B. (**B**) RT-PCR of *GJB1* exon 2 (from g.13615 to g.13943) showed a decreased expression of the patient’s cDNA. A semiquantitative densitometric analysis demonstrated a 70% reduction of transcript level in P when compared to N. (**C**) Both *PMP22* (438 bp, spanning from exon 1 to exon 4) and the housekeeping *GAPDH* (252 bp, spanning from exon 6 to exon 8) cDNAs in patient P had similar expression levels to control N. (**D**) Nucleotide sequence of a normal control: *GJB1* exon 1B and exon 2 are joined together. A vertical line represents the boundary between exon 1B and exon 2. (**E**) Nucleotide sequence of patient III-8: an arrow points to the c.-17+1G>T mutation. The change of the canonical splice site sequence causes the retention of the whole (356 nt-long) intron 1B into the mRNA. Only the first and last 10 nucleotides of intron 1B are shown for a better output.

Using primers spanning intron 1B, the amplification of the sample from patient III-8 (P) resulted in a 487 bp—cDNA fragment, while the normal-control sample (N) showed the expected length of 131 bp (Figure 3A). Sequencing of the latter cDNA confirmed the normal splicing of intron 1B, whereas the patient’s cDNA demonstrated the retention of the entire intron in the amplified product (Figure 3D,E).

RT-PCR was also performed with primers spanning *GJB1* exon 2, and the results underwent a semiquantitative densitometric analysis of corresponding electrophoretic bands to assess transcript levels: mutated samples revealed a 70% reduction of intensity when compared to the normal nerve, while there was no difference in *PMP22* and *GAPDH* levels between patient and control (Figure 3B,C).

### 3.4. Immunofluorescence Study

**Figure 4:**
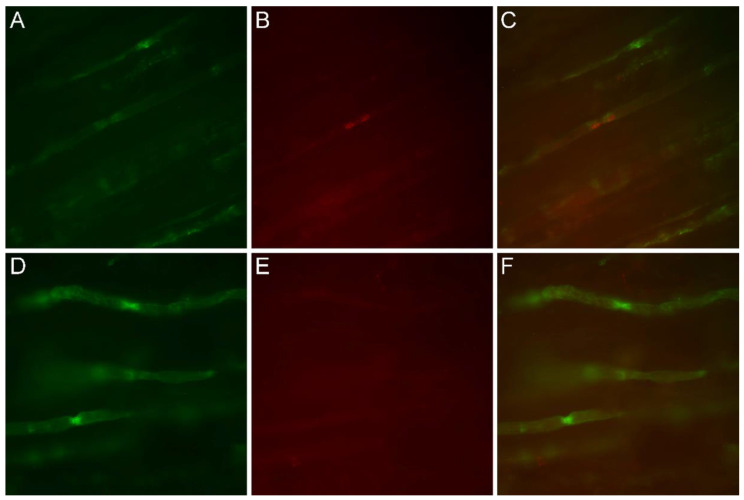
Immunofluorescence study on nerve longitudinal sections, comparing patient III-8 (**D**–**F**) to a normal control (**A**–**C**). Original magnification 40×. (**A**,**D**) Green immunofluorescence following reaction with anti-MAG antibodies. (**B**,**E**) Red immunofluorescence using anti-Cx32 antibodies. (**C**,**F**) Merged images. In the control nerve sample, anti-MAG and anti-Cx32 antibodies colocalized at the paranodes (**C**), whereas in the patient, Cx32 signal was lacking (**E**) and the sole anti-MAG immunofluorescence was observed in merged images (**F**).

Red immunofluorescence confirmed membrane expression of Cx32 at paranodal loops and Schmidt-Lanterman incisures of a control nerve, which also showed MAG colocalization (Figure 4C). On the other hand, the patient’s nerve (III-8) only exhibited the green anti-MAG signal, with no evidence of the presence of Cx32 (Figure 4F).

## 4. Discussion

*GJB1* (chromosome Xq13.1) is the second most common mutated gene in patients with CMT, accounting for up to 10% of patients who are diagnosed with CMTX1 [2,11]. This form of CMT typically presents with a length-dependent sensory-motor neuropathy that usually affects males earlier and more severely than females. Patients frequently develop *pes cavus* with hammer toes and split hands, as well as slowly progressive distal muscular weakness and atrophy (initially involving the lower limbs), along with sensory abnormalities. In a few cases, transient symptoms regarding the central nervous system and reversible white matter lesions are also present. Nerve conduction studies have generally displayed demyelinating features with coexisting signs of axonal damage, with ambivalent neurophysiological values that are consistent with the localization of Cx32 in the noncompact myelin of paranodes and Schmidt-Lanterman incisures [12,13], thus serving as an interface between Schwann cells and axons. Indeed, Cx32 is the most abundant connexin isoform in Schwann cells and it is likely essential for myelin formation [14] and the homeostasis of myelinated axons, providing a shortened radial communication between the abaxonal nucleus of Schwann cell and the adaxonal region by forming channels to transport ions, small metabolites and signalling molecules. The loss of the ability to form these intracellular gap junctions is presumed to be at the basis of the disease [15], considering the similarities between common point mutations and the few reported cases due to the deletion of the entire coding sequence of *GJB1* [16,17,18]. Likewise, some missense variants are known to mediate the loss of *GJB1* function by producing ineffective channels (i.e., with different permeability [19,20]) or by causing mislocation of Cx32 [21], thus impairing the diffusion of messengers and nutrients through gap junctions. Cx32 deficiency could therefore damage normal glial-neuronal interactions that are pivotal in the maintenance of myelin sheaths and axons [22].

However, new insight about the elaborate regulatory components of *GJB1* has suggested new possible pathogenic mechanisms [23], hinting to new perspectives of a still elusive explanation of some phenotypic features. Not only mutants can act as a dominant-negative inhibitor when interacting with other connexin isotypes [24], but *GJB1* also displays a complex control system that normally allows for a specific regulation of expression (responding to particular cellular needs and environmental changes), yet makes the protein vulnerable to further genetic and epigenetic attacks. As a clue to the relevance of its functions, *GJB1* mRNA can be translated with cap-independent mechanisms, and patients with CMTX1 have already been described [25] in association with mutations affecting such regulatory elements (i.e., internal ribosome entry sites, IRES, that usually control the interaction with translation initiating factors, RNA-binding proteins and with the small ribosomal subunit itself). However, most of the regulation of *GJB1* seems to be at the transcription level, and mutations in the 5′ and 3′ UTRs have been recognized as causes of CMTX1 since 1996 [26]. Indeed, while most tissues express multiple connexins, Cx32 is selectively transcripted starting from tissue-specific promoters: P2 activates transcription in the peripheral nervous system, while the central nervous system depends on both P1 and P2 [5]. Consequently, the deletion of the entire P2 sequence (extending from c.-5413 to c.-49) can intuitively impair transcription and thus Cx32 expression in the nerve without any variation of the coding sequence [27]. Binding sites for transcription factors SOX10 and EGR2 are also crucial to activate the promoter and Cx32 production [6,28,29]. Moreover, mutations may involve splicing sites, potentially altering the sequences and the characteristics of the transcript (Figure S1). Still, only few reports have demonstrated the real effects of mutations in 5′ UTR on their transcription to mRNA. For example, Flagiello et al. extracted *GJB1* transcripts from sural nerve biopsies of two CMTX heterozygous females: since only the wild-type allele was detected by retro-transcription of the extracts, it was assumed that the c.-107C>T transition caused the instability of the corresponding transcript [30]. Afterward, Benedetti et al. proved that a c.-16-3C>G substitution activated a cryptic splice site, so that the altered splicing of *GJB1* mRNA resulted in the deletion of the first 278 nucleotides of exon 2 [31]. Likewise, a recent paper reported two male brothers with typical CMTX1 features and the same c.-17+1G>T substitution as our families [6];given the proximity to the known c.-17G>A mutation [6,32], the variant was presumed to exert a similar effect on the splicing of intron 1, but no pathogenetic mechanism was established. When compared to that report, our patients also had fairly typical clinical, neurophysiological and pathological features, bearing a phenotype that cosegregated with the variant. The transversion changed the first nucleotide of intron 1B and was predicted to abolish the canonical donor splice site by dedicated algorithms. This hypothesis was confirmed here by the evidence of a transcript that was 356 bp longer than the mRNA of a control nerve biopsy, thus demonstrating the retention of the entire intronic sequence 1B.

RT-PCR also suggested that this mutation could lead to a loss of function pathogenetic mechanism. Indeed, there was a marked reduction of the transcript corresponding to exon 2 when compared to the wild-type sample. However, this scarcity could not be secondary to myelin fibre loss in the sampled tissue, since the expression of the myelin gene *PMP22* as well as of the housekeeping *GAPDH* retained normal levels. Moreover, immuno-microscopy did not show Cx32 fluorescent signal in the patient’s nerve, replicating the same pattern as *GJB1* missense mutations that lack regular gap junctions.

These results are consistent with previous studies concerning fine regulation of *GJB1*: its proper expression requires the precise processing of the correct order of the polypeptide chain just as much as of the sequences that regulate the transcript’s length and subsequent activation of translation. Indeed, in our patients, aberrant splicing in the 5′ UTR ended in the loss of Cx32, as previously hypothesized for similar noncoding variants [30,32] that reportedly affect transcript stability. Less likely, the mutation could exert a direct effect on protein translation by hindering the recognition of the nearby start codon and its accessibility to the ribosome. Also, our results cannot entirely exclude the possibility of the mutation uncovering an upstream start codon in the intronic sequence with a potential reading frameshift, similarly to what Sargiannidou confuted in regard to the p.Met1Ile start codon mutation [33].

## 5. Conclusions

We demonstrated that the candidate variant c.-17+1G>T—which was first identified in a previous paper [6]—does cause CMTX1. The mutation cosegregates with typical clinical phenotype in two unrelated Italian pedigrees and is located in a highly conserved position, at the interface between intron 1B and exon 2, like other adjacent putative causative variants [31,32], thus emphasizing its functional importance. In agreement with mapping algorithms, the transversion disrupts splice-site consensus sequences. The complete retention of intron 1B generates a longer and likely unstable transcript that results in loss of Cx32, as proven by retro-transcriptional and selective immunofluorescence studies on a patient’s archived nerve biopsy. The experimental work also remarks the role of 5′UTR of *GJB1* in the pathogenesis of CMTX1 and highlights the need to include this region in the routine screening, as well as in NGS panels for patients with consistent clinical and familiar clues.

## Figures and Tables

**Table 1:** Summary of the main clinical features of the patients. Abbreviation: CMTES = Charcot-Marie-Tooth Examination Score; M = male; F = female; y.o. = year old; MRC = Medical Research Council Scale for muscle strength; cMAP = compound motor action potential; MCV = motor conduction velocity; SAP = sensory action potential; SNCV = sensory nerve conduction velocities.

	Patient (CMTES)	Gender	Onset	Main Symptoms/Signs	Nerve Conduction Studies	Nerve Biopsy
1st family (Figure 1A)	II-2 (12)	M	II decade (hand tremor and cramps)	54 y.o.: Stepping gait (needing ankle-foot-orthoses) with peroneal hypotrophy and areflexia. *Pes equinovarus* with *griffe* of toes; split hand. Distal and severe hypopallesthesia with ataxic gait. Cramps at rest and during exercise. Mild postural tremor of upper limbs. Bilateral hypoacusia.	17 and 54 y.o.: Severe reduction of cMAP of peroneal (1.5 mV → not evokable) and then median nerves (0.6 mV) with progressive decrease in conduction velocities (44 m/s and 29 m/s, respectively). Preserved sural nerve: SAP = 9 μV; SNCV = 27 m/s.	17 y.o.: Loss of large nerve fibers, rare regeneration clusters (Figure 2A)
	II-1 (13)	M	II decade (walking and running difficulties since he was 10)	22 y.o.: Stepping gait with peroneal hypotrophy and weakness, lower limbs’ areflexia and distal sensory loss (feet apallesthesia) with ataxia and deficient proprioception; underwent surgery because of *pes cavus* at 15 years of age. Upper limbs: tremor and progressive weakness since he was 17; hyporeflexia; hand and forearm muscular hypotrophy.		
	I-2 (3)	F	V decade (mild walking difficulties)	44 y.o.: Bilateral *pes cavus*, mild weakness in foot plantar flexion (while walking on toes) 84 y.o.: Still paucisymptomatic		
2nd family (Figure 1B)	III-9 (4)	F	IV decade (mild walking difficulties)	33 y.o.: *Pes cavus*; mild weakness in hallux and foot dorsiflexion (MRC 4+/5); stocking-like sensory loss; preserved deep tendon reflexes and muscle trophism	38 y.o.: Reduction of peroneal cMAP (2 mV); non-evokable sural SAP; intermediate motor conduction velocities (37 m/s for both peroneal and median nerves, 40 m/s for ulnar nerve).	
	III-10 (11)	M	III decade (walking difficulties and progressive distal atrophy)	28 y.o.: Stepping gait with ankle-foot orthosis; loss of deep tendon reflexes; *pes equinovarus*; “stoking and glove” deep sensory loss; ataxia. Simian hand. Upper limb postural tremor.		15 y.o.: Axonal neuropathy, mainly affecting large fibers; signs of regeneration
	III-8 (8)	M	II decade (walking difficulties)	18 y.o.: Stepping gait with lower limbs’ distal hypotrophy and areflexia; bilateral *pes cavus.* Preserved strength and deep tendon reflexes on upper limbs.	17 y.o.: Reduction of peroneal cMAP (2 mV); decrease in motor conduction velocities (32 m/s for peroneal nerve, 36 m/s for median nerve)	18 y.o.: Axonal neuropathy with moderate reduction of large nerve fibers; sparse regeneration clusters of small fibers (Figure 2B)
	I-2; II-2; II-4	F	N/A	Mild walking difficulties		

## Data Availability

The data presented in this study are available in the supplementary materials. Further details are available on request from the corresponding author.

## Supplementary Materials

The following are available online at https://www.mdpi.com/2076-3425/11/1/24/s1, Figure S1: *GJB1* gene structure and schematic drawing of the experimental design, highlighting the different length of splice variants, Figure S2: Sequencing chromatograms illustrating the results of GJB1 genetic testing in some representative family members, Table S1: List of used primers.
