# Supplementary material for: Aberrant Splicing in GJB1 and the Relevance of 5′ UTR in CMTX1 Pathogenesis
Source: Brain Sci. 2020 Dec 27;11(1):24. doi: 10.3390/brainsci11010024 (PMC7824018; doi:10.3390/brainsci11010024)
Supplement: Supplementary file 1 [file brainsci-11-00024-s001.pdf]

Supplementary Materials

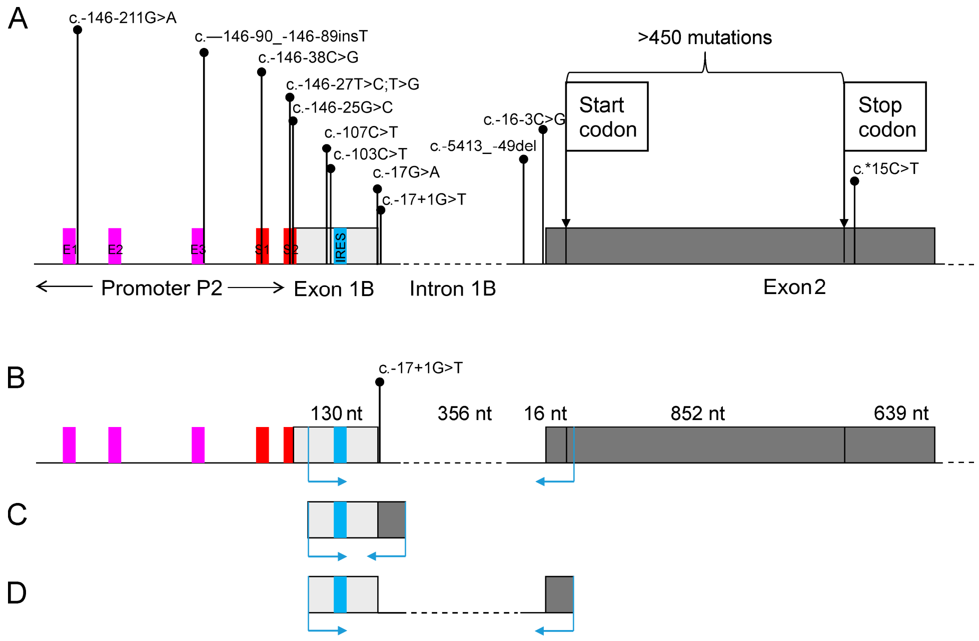

Figure S1. *GJB1* gene structure and schematic drawing of the experimental design, highlighting the differ-ent length of splice variants. (a) Gene structure and known mutations in the untranslated regions of *GJB1*. Pink boxes E1-E3 represent EGR2 binding sites, while red boxes S1-S2 mark SOX10 binding sites. Internal Ribosomal Entry Site (IRES) is highlighted by the homonym light blue box. (b) Experimental design: a couple of primers (GJB1-ex1B_1 Fw, GJB1-5'UTR_3 Rv) was used to amplify *GJB1* noncoding cDNA in order to assess the effect of the c.-17+1G>T mutation. (c) Regular splicing: cor-rect removal of intron 1B generates a cDNA fragment of expected 131 nucleotides in wild type samples. (d) Transcrip-tional analysis in mutant samples: since c.-17+1G>T mutation abolishes a donor splice-site consensus sequence, intron 1B should be retained in its complete length, thus producing a longer fragment of cDNA.

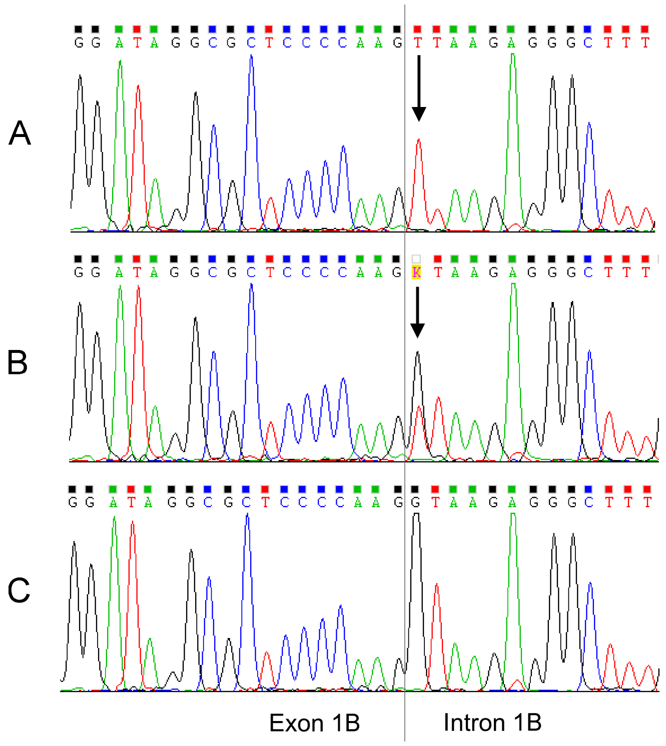

Figure S2. Sequencing chromatograms illustrating the results of GJB1 genetic testing in some representa-tive family members. (A) a hemizygous CMTX1 male (II-2, first family); (B) a heterozygous CMTX1 female (III-9, second family); (C) a healthy individual (II-4, first family). An arrow points to the c.-17+1G>T mutation.

Table S1. List of used primers. After exclusion of mutations in *GJB1* exon 2 coding sequence, 5′UTR was sequenced from nt g.12671 to g.13503. Nucleotide numbering was in relation to NG_008357.1, covering *GJB1* transcript NM_000166.5. *PMP22* and *GAPDH* nucleotide numberings were according to NM_000304.3 and NM_002046.5, respectively. Primer pairs GJB1-5′UTR_1, GJB1-5′UTR_2 and GJB1-5′UTR_3 were used to sequence 5′UTR genomic DNA, while primer pairs GJB1-ex2 were used to amplify cDNA coding sequence. GJB1-ex1B_1 was used with reverse primer GJB1-5′UTR_3 to amplify and sequence 5′UTR cDNA; GJB1-ex1B_2 was used in nested sequencing on the previous PCR amplification product.

Primers pair name	Position (first nt; last nt of the amplicon)	Forward sequence	Reverse sequence
GJB1-5′UTR_1	g.12671; g.12993	taagcttctgacggggccat	ctgctttatacccagtgtctgc
GJB1-5′UTR_2	g.12907; g.13219	gaagtcagggcgtttgatct	gcccatcttgttccgatatcag
GJB1-5′UTR_3	g.13139; g.13503	ttgagtttgccccaggtctg	agttcatcctgcctcattcacac
GJB1 ex2	g.13615; g.13943	cagagagtgtgtggggtgatg	gaagacggcctcaaacaacag
GJB1-ex1B_1	g.13017	ggtgatgaattgggacgca	
GJB1-ex1B_2	g.13078	ccataggggacctgtctggg	
PMP22 ex1-4	c.21; c.458	atcatcgtcctccacgtcg	atagatgacaccgctgagaa
GAPDH ex6-8	c.386; c.636	ccatgttcgtcatgggtgtg	gccagtagaggcagggatg
